# Lepromatous leprosy, melanoma, and basal cell carcinoma: clinical-histopathologic association^☆^^☆☆^

**DOI:** 10.1016/j.abd.2019.09.008

**Published:** 2019-09-30

**Authors:** Cintia Santos Braghiroli, Maria Rita Parise-Fortes, Mariângela Esther Alencar Marques, Joel Carlos Lastória

**Affiliations:** a Department of Dermatology and Radiotherapy, Faculdade de Medicina de Botucatu, Universidade Estadual Paulista, Botucatu, SP, Brazil; b Department of Pathology, Faculdade de Medicina de Botucatu, Universidade Estadual Paulista, Botucatu, SP, Brazil

**Keywords:** Carcinoma, basal cell, Leprosy, Melanoma

## Abstract

Cutaneous neoplasms frequently occur in leprosy, but there are few reports of the coexistence of leprosy and basal cell carcinoma in the same lesion. This case reports a 49-year-old male with an ulcerated plaque on the right lateral nasal wall, bright papules on the sternal region, and a blackened plaque on the right temporal region. The nasal and temporal lesions were diagnosed by histopathology as basal cell carcinoma and melanoma, respectively. The sternal lesions were excised with the repair of the “dog ear” which histopathological examination showed macrophages in the dermis parasitized with acid-fast bacilli, confirming the diagnosis of lepromatous leprosy with Fite-Faraco staining. This case report highlights the importance of referring the dog-ear specimen for histopathologic analysis.

## Introduction

Basal cell carcinoma (BCC) is one of the most common malignant skin tumors, accounting for about 75% of all skin cancers, most commonly manifested on sun-exposed skin such as the head and neck of older individuals.^1^, ^2^ Melanoma is the most aggressive cutaneous malignancy and represents 10% of all skin cancer diagnosed.^3^ Melanoma is less common but more aggressive than BCC.^4^

Leprosy is a chronic infectious disease caused by *Mycobacterium leprae*, an intracellular parasite that mainly affects skin and peripheral nerves, with tropism for macrophages and Schwann cells. The disease is transmitted through prolonged contact with untreated lepromatous leprosy patients.^5^, ^6^ The coexistence of BCC and leprosy in the same lesion is uncommon, but it has been previously documented.^7^

In the present report, the diagnosis of leprosy was made through a histopathological finding in the dog-ear fragment excised during the surgical removal of a BCC. The authors describe the triple association of BCC, lepromatous leprosy, and melanoma in a male patient with no known immunodeficiency.

## Case report

**Figure 1:**
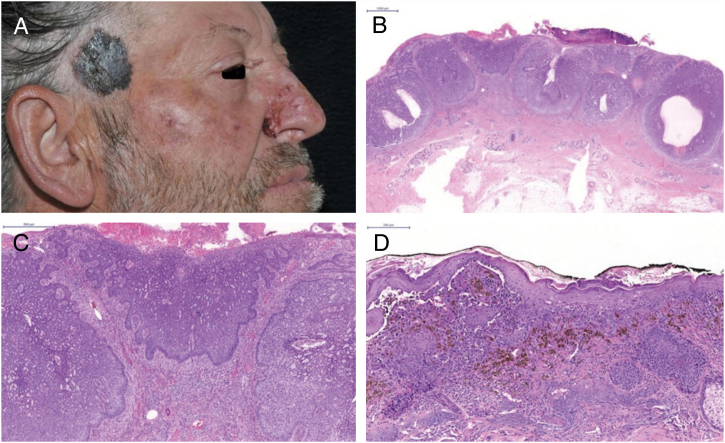
(A) Basal cell carcinoma (BCC) – nasal; melanoma – right temporal region. (B) BCC – clusters of basaloid cells (Hematoxylin & eosin, ×10). (C) Peripheral palisading of cells (Hematoxylin & eosin, ×100). (D) Melanoma – nests of atypical melanocytes and pagetoid spread (Hematoxylin & eosin, ×200).

**Figure 2:**
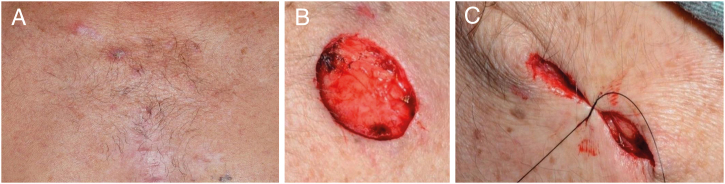
(A) Basal cell carcinomas (BCCs) on the sternal region; (B) surgical excision; (C) synthesis and “dog ear.”.

**Figure 3:**
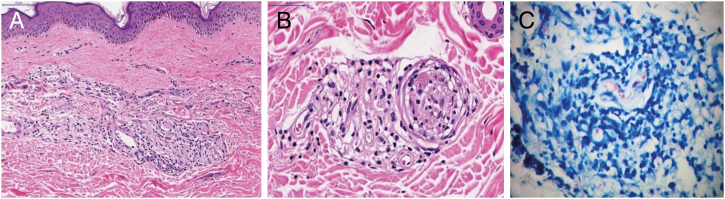
Histopathology of the “dog ear”: (A) inflammatory infiltrate in peri-adnexal superficial and deep dermis, lymphocytes and macrophages with vacuolated cytoplasm (Hematoxylin & eosin, ×400). (B) Perineurium delamination and infiltration by lymphocytes and macrophages (Hematoxylin & eosin, ×400). (C) Acid-fast bacillus positive with intact granular bacilli and globus formation (Fite-Faraco, ×1000).

The patient was a 49-year-old male gardener with a large ulcerated lesion on the right lateral nasal wall for three years and on the sternal region for five years. The sternal lesion showed erythematous papules and a shiny surface, while the nasal lesion was a scar-like plaque with papules on the surface, meliceric crusts, and ulcerations. There was also a blackish plaque on the right temporal region, measuring approximately 4 cm. Dermoscopy revealed a blue-white veil, chrysalis, globules, and irregular streaks on the periphery. Incisional biopsies were performed on the nasal and right temporal lesions, confirming ulcerated nodular BCC and melanoma, respectively (Fig. 1). The sternal lesion was completely resected by elliptical excision, with the need to correct the “dog ear,” which was also referred for histopathologic analysis (Fig. 2). The lesion was consistent with BCC, and the fragment from the “dog ear” showed some alterations that led to the need for acid-fast bacillus (AFB) staining, which revealed the presence of numerous intact granular bacilli with globus formation, resulting in the diagnosis of multibacillary leprosy (Fig. 3). The patient presented ciliary madarosis, rarefaction of the terminal eyebrows, thickening of the skin on the frontal region and ears, and bilateral thickening of the ulnar nerve. The patient was treated for lepromatous leprosy with multidrug therapy (MDT: dapsone, rifampicin, and clofazimine) and total excision of the nasal and temporal lesions was performed. Histologic analysis of the melanoma demonstrated the vertical growth phase, with Breslow thickness of 1.2 mm and Clark level IV.

## Discussion

The authors describe the co-occurrence of BCC and melanoma in a patient with lepromatous leprosy, whose diagnosis was made through histopathologic analysis of the skin fragment from the “dog ear” excised during surgical removal of the BCC.

BCC is one of the most prevalent tumors, and exposure to UV radiation is the main risk of factor for the development of these tumors. The immune system is fundamental in the prevention and control of skin tumors, and the development appears to be directly linked to immunosuppression caused by the cumulative effect of UV radiation, which acts to suppress the local and systemic immune response.^8^

Malignancy developing in trophic ulcers in patients with lepromatous leprosy (*e.g.*, squamous cell carcinoma and nodular melanoma) is extremely rare, with only a few cases reported in the literature.^9^, ^10^

There is no report on whether immunosuppression in patients with lepromatous leprosy favors a more aggressive spread of malignant lesions. As the immune response is specific to *M. leprae*, it is suggested that this factor cannot be associated with the development of malignant tumors or susceptibility to other pathogens.

The present case suggests that the coexistence of *M. leprae* and skin tumors in the same lesion is probably secondary to large numbers of bacilli, although no bacilli were found in the marginal analysis of the fragments from the excised lesions on the nasal and temporal regions.

The patient presented clinical signs of lepromatous leprosy, such as ciliary madarosis and terminal rarefaction of the eyebrows, infiltration of the frontal region and ears, and bilateral thickening of the ulnar nerve. These clinical signs of leprosy should have been observed before the skin tumors, since the diagnosis of the disease is based on clinical symptoms. Early diagnosis and specific treatment are essential to interrupt disease transmission.

This case report shows the importance of complete dermatological examination and also reports the association between leprosy and cutaneous malignancies, which is still poorly understood.

## Funding

None declared.

## Author's contribution

Cintia Santos Braghiroli: Iintellectual participation in propaedeutic and/or therapeutic conduct of the cases studied.

Maria Rita Parise-Fortes: Obtaining, analyzing and interpreting the data.

Mariângela Esther Alencar Marques: Obtaining, analyzing and interpreting the data.

Joel Carlos Lastória: Intellectual participation in propaedeutic and/or therapeutic conduct of the cases studied.

## Conflicts of interest

The authors declare no conflicts of interest.
